# Coronary heart disease increases the risk of perioperative ischemic stroke after noncardiac surgery: A retrospective cohort study

**DOI:** 10.1111/cns.14912

**Published:** 2024-08-26

**Authors:** Rui Wang, Han Wang, Siyuan Liu, Lujia Yang, Libin Ma, Fengjin Liu, Yingfu Li, Peng Li, Yizheng Shi, Miao Sun, Yuxiang Song, Wugang Hou, Weidong Mi, Yulong Ma

**Affiliations:** ^1^ Department of Anesthesiology The First Medical Center of Chinese PLA General Hospital Beijing China; ^2^ Nation Clinical Research Center for Geriatric Diseases Chinese PLA General Hospital Beijing China; ^3^ Department of Orthopedics Air Force Medical Center Beijing China; ^4^ Department of Anesthesiology Affiliated Hospital of Nantong University Nantong China; ^5^ Department of Emergency Yantai Yuhuangding Hospital Yantai China; ^6^ Department of Anesthesiology The Sixth Medical Center of Chinese PLA General Hospital Beijing China; ^7^ Department of Anesthesiology and Perioperative Medicine Xijing Hospital, Air Force Military Medical University Xi’an China

**Keywords:** coronary heart disease, noncardiac surgery, perioperative ischemic stroke, preoperative mean arterial pressure, propensity score matching

## Abstract

**Objective:**

To investigate the association between coronary heart disease (CHD) and the risk of perioperative ischemic stroke in patients undergoing noncardiac surgery.

**Methods:**

This retrospective study evaluated the incidence of ischemic stroke within 30 days after a noncardiac surgery. A cohort of 221,541 patients who underwent noncardiac surgery between January 2008 and August 2019 was segregated according to whether they were diagnosed with CHD. Primary, sensitivity, and subgroup logistic regression analyses were conducted to confirm that CHD is an independent risk factor for perioperative ischemic stroke. Propensity score matching analysis was used to account for the potential residual confounding effect of covariates.

**Results:**

Among the 221,541 included patients undergoing noncardiac surgery, 484 patients (0.22%) experienced perioperative ischemic stroke. The risk of perioperative ischemic stroke was higher in patients with CHD (0.7%) compared to patients without CHD (0.2%), and multivariate logistic regression analysis showed that CHD was associated with a significantly increased risk of perioperative ischemic stroke (odds ratio (OR), 3.7943; 95% confidence interval (CI) 2.865–4.934; *p* < 0.001). In a subset of patients selected by propensity score matching (PSM) in which all covariates between the two groups were well balanced, the association between CHD and increased risk of perioperative ischemic stroke remained significantly significant (OR 1.8150; 95% CI, 1.254–2.619; *p* = 0.001). In the subgroup analysis stratified by age, preoperative β‐blockers, and fibrinogen‐to‐albumin ratio (FAR), the association between CHD and perioperative ischemic stroke was stable (*p* for interaction >0.05). Subgroup analyses also showed that CHD was significantly increased the risk of perioperative ischemic stroke in the preoperative mean arterial pressure (MAP) ≥94.2 mmHg subgroups (*p* for interaction <0.001).

**Conclusion:**

CHD is significantly associated with an increased risk of perioperative ischemic stroke and is an independent risk factor for perioperative ischemic stroke after noncardiac surgery. Strict control of preoperative blood pressure may reduce the risk of perioperative ischemic stroke for patients with CHD undergoing noncardiac surgery.

## Graphical abstract

CHD was identified as an independent risk factor for perioperative ischemic stroke in our large retrospective cohort study of noncardiac surgery patients. Strict control of preoperative blood pressure may reduce the risk of perioperative ischemic stroke in patients with CHD.

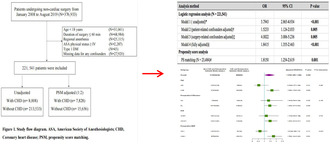

## 1 INTRODUCTION

Perioperative ischemic stroke is a serious neurological complication that occurs within 30 days after surgery. The incidence of perioperative ischemic stroke after cardiovascular surgery is relatively high, occurring in between 1.9% and 9.7% of patients,^1^ whereas the incidence is much lower, between 0.1% and 1.9%, after noncardiac, non‐neurological, and nonmacrovascular surgeries.^2^, ^3^ Perioperative stroke is associated with poor prognosis, higher mortality, and disability because most patients do not receive thrombolytic therapy, either due to delayed diagnostic imaging or a high risk of bleeding after surgery.^4^, ^5^, ^6^, ^7^, ^8^ A high number of perioperative factors and patient comorbidities result in a complex and multifactorial etiopathogenesis of perioperative ischemic stroke,^9^ reported risk factors include advanced age, previous stroke or transient ischemic attack (TIA), renal disease, and vascular disease or propagation of vascular disease.^2^, ^3^, ^10^ To improve clinical outcomes, the field requires more precise methods to recognize patients at risk of ischemic stroke during perioperative period as well as protocols to intervene quickly when a patient is suspected of suffering one of these events.

Previous studies have reported that patients with myocardial infarction, cardiac valvular disease, atrial fibrillation, or congenital heart disease have a higher risk of experiencing ischemic stroke.^2^, ^3^, ^11^ Coronary heart disease (CHD) is the most common heart disease and is related to worse functional outcomes and increased mortality after ischemic stroke.^12^, ^13^, ^14^ On this basis, CHD is considered as a risk factor for perioperative stroke^15^; however, whether CHD increases the risk of perioperative ischemic stroke has not been well established empirically.

Herein, we conducted a retrospective study of 221,541 patients undergoing noncardiac surgery and screened 8008 patients with CHD to examine the relationship between CHD and the risk of perioperative ischemic stroke.

## 2 METHODS

### 2.1 Study design and population

This research protocol was approved by the Medical Ethics Committee of Chinese PLA General Hospital (reference number: S2021‐493‐01), and the requirement for written informed consent was waived. This manuscript adheres to the applicable Strengthening the Reporting of Observational Studies in Epidemiology (STROBE) guidelines (Table S1).

**FIGURE 1:**
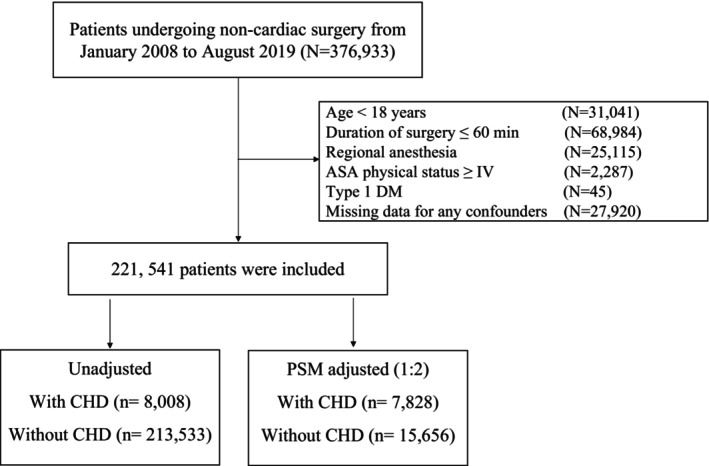
Study flow diagram. ASA, American Society of Anesthesiologists; CHD, coronary heart disease; PSM, propensity score matching.

In this retrospective cohort study, we included patients aged ≥18 years old, who underwent noncardiovascular surgery, between January 1, 2008 and August 31, 2019 at the First Medical Center of Chinese PLA General Hospital, a tertiary referral academic hospital in Beijing, China. The screening protocol to identify eligible patients is depicted in Figure 1. We excluded patients with: (1) American Society of Anesthesiologists (ASA) physical status ≥IV, (2) duration of surgery ≤60 min, (3) type 1 diabetes mellitus (DM), (4) procedures that required only regional anesthesia, and (5) missing data for any confounders. For patients who had more than one surgery during the study period, only the first qualifying surgery was included in the analysis. Perioperative ischemic stroke is defined as ischemic cerebral infarction associated with sensory, motor, or cognitive impairments (such as hemiplegia, aphasia, sensory, and memory impairments) within 30 days after surgery.^3^, ^16^, ^17^ Patients were identified as experiencing a perioperative ischemic stroke if a qualifying, identified through ICD‐9/10 diagnosis code was assigned to the patient within 30 days after the surgical procedure (Table S2).

### 2.2 Definitions of variables and data collection

The exposure of interest was assignment of a qualifying ICD‐9/10 diagnostic code for CHD before the perioperative ischemic stroke event. The primary outcome was the perioperative ischemic stroke within 30 postoperative days.

We defined 26 potential confounders in this study. Among these, 16 were patient‐related confounders, including sex, age, ASA class, body mass index (BMI), preoperative β‐blockers, preoperative hemoglobin (Hb), preoperative albumin (ALB), preoperative total bilirubin (TBIL), preoperative prothrombin time (PT), preoperative neutrophil‐to‐lymphocyte ratio (NLR), preoperative plasma fibrinogen (PF), preoperative platelet‐to‐lymphocyte ratio (PLR), and preoperative fibrinogen‐to‐albumin ratio (FAR), chronic kidney disease, arrhythmia, and chronic obstructive pulmonary disease (COPD). The other confounders were surgery‐related, including surgery type, surgery length, emergency surgery, estimated blood loss, malignant tumor, colloids infusion, crystalloids infusion, preoperative mean arterial pressure (MAP), morphine equivalents, and blood products depot.

### 2.3 Statistical analysis

To control for potential confounding effects, we conducted a logistic regression analysis to assess the relevance of CHD to perioperative ischemic stroke. We built four models: (1) Model 1 was an unadjusted univariable model, (2) Model 2 was used to adjust for the aforementioned patient‐related confounders, (3) Model 3 was used to adjust for the aforementioned surgery‐related confounders, and (4) Model 4 was the fully adjusted model that included all 26 identified confounders (Table 2).

To further validate the correlation between CHD and the risk of perioperative ischemic stroke, a 2:1 non‐CHD:CHD propensity score matching (PSM) was conducted using a logistic regression model and the following covariates: BMI, age, ASA class, preoperative β‐blockers, and surgery type. After obtaining the matched or weighted data, standardized mean difference (SMD) and Kernel density plots were used to evaluate the balance of covariates among the two groups. An SMD <0.2 is considered as acceptable deviation for a covariate.^18^, ^19^ The association between CHD and perioperative ischemic stroke was estimated using logistic regression analysis.

Advanced age, β‐blockers, FAR, and preoperative MAP were associated with the risk of CHD complications in several previous studies.^20^, ^21^, ^22^, ^23^ Thus, we performed subgroup analysis according to patient status for advanced age (≥60 years), β‐blockers, FAR (continuous variables are converted into binary categories with median as cutoff value), and preoperative MAP (continuous variables are converted into binary categories with median as cutoff value). Consequently, we conducted subgroup analyses based on these factors, using unadjusted variables with Bonferroni correction for multiple comparisons.

A two‐sided *p* value <0.05 indicated statistical significance. Statistical analyses were performed using R (version 1.4.1106, R Foundation for Statistical Computing, Vienna, Austria), along with tableone, MatchIt, Matching, Cobalt, rms, and car.

## 3 RESULTS

### 3.1 Baseline patient characteristics

**FIGURE 2:**
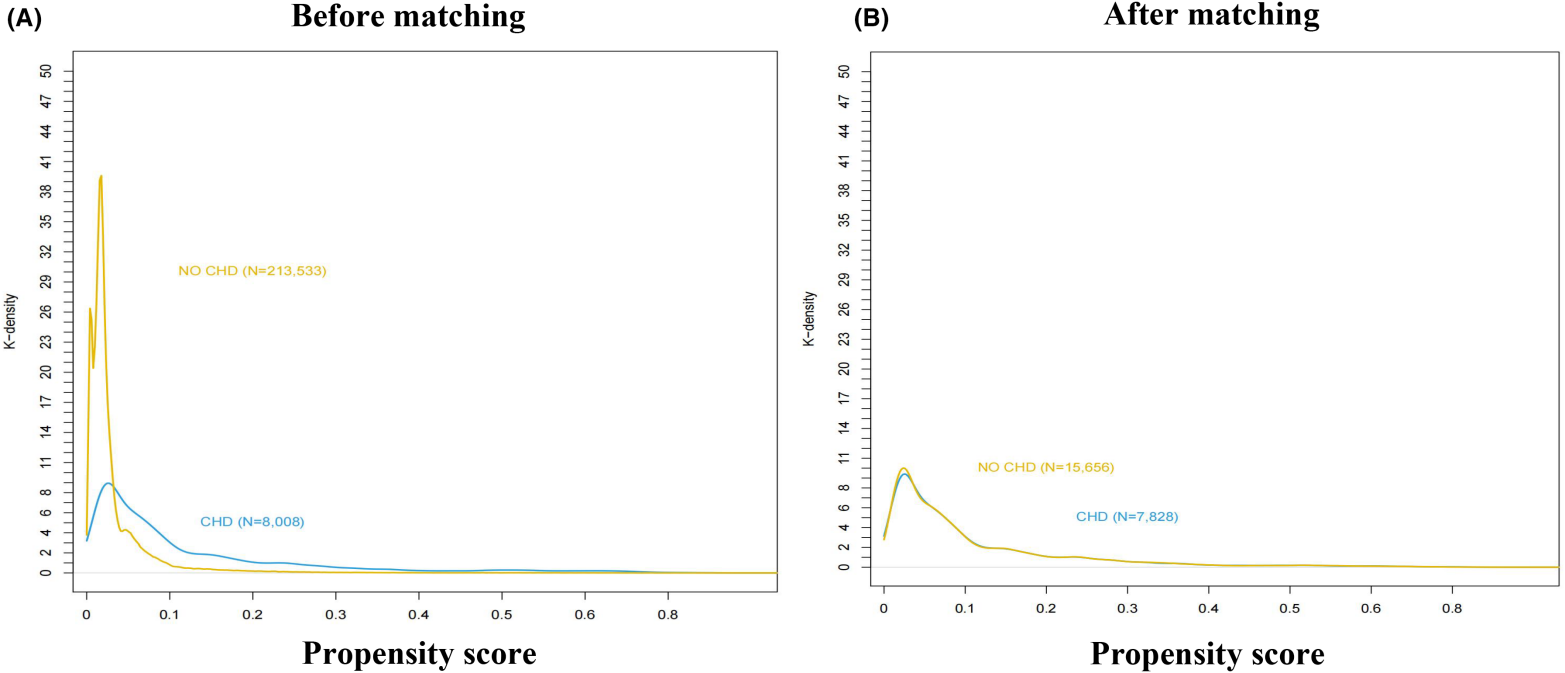
The propensity score histograms of the two groups. (A) Before matching. (B) After matching. CHD, coronary heart disease.

This study evaluated 221,541 patients who underwent noncardiac surgery at Chinese PLA General Hospital between January 1, 2008 and August 31, 2019. The study population was divided into two groups according to whether they were diagnosed with CHD (CHD group; 8008 patients) or not (non‐CHD group; 213,533 patients). Baseline characteristics are summarized in Table 1. Patients in the CHD group had higher BMI and more malignant tumor surgery than that in the non‐CHD group. Compared with patients in the non‐CHD group, those in the CHD group were more likely to have a history of arrhythmia, COPD, and chronic kidney disease. Patients in the CHD group were also more likely to undergo intraperitoneal surgery or thoracic surgery, whereas patients in the non‐CHD group were more likely to undergo neurosurgery. After adjustment using propensity score matching (PSM), all covariates were well balanced between groups (Table 1 and Figure 2).

**TABLE 1:** Baseline characteristics unadjusted sample and propensity score‐matched sample.

Characteristic	Unadjusted sample (*N* = 221,541)			PSM adjusted (2:1) (*N* = 23,484)		
	Patients without CHD (*n* = 213,533)	Patients with CHD (*n* = 8008)	SMD	Patients without CHD (*n* = 15,656)	Patients with CHD (*n* = 7828)	SMD
Age, years						
[18, 65]	176,973 (82.9)	3898 (48.7)	0.78	7501 (47.9)	3894 (49.7)	0.043
[65, 75]	29,110 (13.6)	2910 (36.3)		5887 (37.6)	2796 (35.7)	
[75, 81]	5950 (2.8)	906 (11.3)		1739 (11.1)	857 (10.9)	
[81, 100]	1500 (0.7)	294 (3.7)		529 (3.4)	281 (3.6)	
Female sex (%)	107,597 (50.4)	3290 (41.1)	0.172	6776 (43.3)	3216 (41.1)	0.045
BMI, kg/m^2^	24.2 [21.8, 26.6]	25.2 [23.0, 27.5]	0.276	25.10 [22.86, 27.68]	25.12 [23.03, 27.44]	0.018
ASA physical status (%)						
Class I	32,319 (15.1)	168 (2.1)	0.762	253 (1.6)	168 (2.1)	0.047
Class II	165,421 (77.5)	5340 (66.7)		10,912 (69.7)	5333 (68.1)	
Class III	15,793 (7.4)	2500 (31.2)		4491 (28.7)	2327 (29.7)	
COPD (%)	1585 (0.7)	151 (1.9)	0.101	226 (1.4)	149 (1.9)	0.036
Arrhythmia (%)	32,102 (15.0)	1921 (24.0)	0.227	2978 (19.0)	1852 (23.7)	0.113
Chronic kidney disease (%)	1844 (0.9)	196 (2.4)	0.124	298 (1.9)	191 (2.4)	0.037
Preoperative Hb, g/L	134.0 [122.0, 146.0]	132.0 [120.0, 144.0]	0.125	133.00 [121.00, 145.00]	132.00 [121.00, 144.00]	0.054
Preoperative ALB, g/L	41.5 [39.1, 43.9]	40.7 [38.2, 43.0]	0.231	40.70 [38.10, 43.20]	40.70 [38.20, 43.10]	0.012
Preoperative TBIL, μmol/L	10.7 [8.0, 14.4]	10.6 [8.0, 14.2]	0.041	10.90 [8.20, 14.60]	10.60 [8.00, 14.20]	0.01
Preoperative NLR	1.8 [1.4, 2.5]	2.0 [1.5, 2.7]	0.052	1.97 [1.46, 2.78]	1.97 [1.49, 2.71]	0.05
Preoperative PLR	116.6 [91.6, 151.8]	116.9 [91.4, 154.5]	0.009	118.59 [92.30, 156.99]	116.90 [91.42, 154.52]	0.051
Preoperative PT, s	13.1 [12.6, 13.6]	13.1 [12.6, 13.7]	0.08	13.10 [12.60, 13.70]	13.10 [12.60, 13.70]	0.001
Preoperative PF	3.0 [2.6, 3.7]	3.3 [2.8, 4.0]	0.284	3.26 [2.77, 3.94]	3.32 [2.82, 4.00]	0.05
Preoperative FAR	0.07 [0.06, 0.09]	0.08 [0.07, 0.10]	0.293	0.08 [0.07, 0.10]	0.08 [0.07, 0.10]	0.032
Preoperative β blockers (%)	6339 (3.0)	1828 (22.8)	0.62	3008 (19.2)	1648 (21.1)	0.046
Emergency (%)	5293 (2.5)	176 (2.2)	0.019	446 (2.8)	174 (2.2)	0.004
Surgery type (%)						
Orthopedic surgery	32,789 (15.4)	1279 (16.0)	0.365	2517 (16.1)	1246 (15.9)	0.038
Intraperitoneal surgery	54,185 (25.4)	2732 (34.1)		5433 (34.7)	2658 (34.0)	
Gynecologic surgery	15,155 (7.1)	193 (2.4)		327 (2.1)	191 (2.4)	
Oral surgery	9257 (4.3)	182 (2.3)		327 (2.1)	182 (2.3)	
Neurosurgical surgery	19,535 (9.1)	341 (4.3)		628 (4.0)	341 (4.4)	
Thoracic surgery	14,498 (6.8)	748 (9.3)		1526 (9.7)	733 (9.4)	
Other (Urological surgery, etc.)	68,114 (31.9)	2533 (31.6)		4898 (31.3)	2477 (31.6)	
Malignant tumor surgery (%)	96,023 (45.0)	4551 (56.8)	0.239	8575 (54.8)	4433 (56.6)	0.037
Surgery length, min	148.0 [100.0, 215.0]	152.0 [109.0, 212.2]	0.006	148.00 [100.00, 215.00]	152.00 [109.00, 212.25]	0.038
Preoperative MAP, mmHg	91.3 [83.3, 99.3]	95.3 [88.0, 103.0]	0.359	95.00 [87.33, 102.67]	95.33 [88.00, 103.00]	0.033
Estimated blood loss, mL						
[−1200]	163,754 (76.7)	6299 (78.7)	0.079	11,904 (76.0)	6151 (78.6)	0.079
[200, 400]	23,362 (10.9)	909 (11.4)		1830 (11.7)	892 (11.4)	
[400, 800]	16,862 (7.9)	538 (6.7)		1220 (7.8)	529 (6.8)	
>800	9555 (4.5)	262 (3.3)		702 (4.5)	256 (3.3)	
Blood products depot (%)	24,235 (11.3)	956 (11.9)	0.018	2153 (13.8)	930 (11.9)	0.056
Autologous blood depot (%)	5340 (2.5)	192 (2.4)	0.007	369 (2.4)	187 (2.4)	0.002
Colloids infusion, mL/kg/min						
[0, 1]	71,801 (33.6)	2474 (30.9)	0.166	4459 (28.5)	2395 (30.6)	0.092
[1, 4]	95,444 (44.7)	4196 (52.4)		8015 (51.2)	4116 (52.6)	
[4, 40]	46,288 (21.7)	1338 (16.7)		3182 (20.3)	1317 (16.8)	
Crystalloids infusion, mL/kg/min						
[0, 7]	84,460 (39.6)	3066 (38.3)	0.027	6352 (40.6)	2998 (38.3)	0.049
[7, 10]	62,004 (29.0)	2399 (30.0)		4620 (29.5)	2351 (30.0)	
[10, 71]	67,069 (31.4)	2543 (31.8)		4684 (29.9)	2479 (31.7)	
Morphine equivalents, mg^a^	120.0 [90.0, 150.0]	135.0 [105.0, 165.0]	0.106	135.00 [105.00, 165.00]	135.00 [105.00, 165.00]	0.037

*Note*: The data are shown as the median (interquartile range), *n* (%), or mean ± SD.

Abbreviations: ALB, albumin; ASA, American Society of Anesthesiologists; BMI, body mass index; CHD, coronary heart disease; COPD, chronic obstructive pulmonary disease; FAR, fibrinogen to albumin ratio; Hb, hemoglobin; MAP, mean arterial pressure; NLR, neutrophil‐lymphocyte ratio; PF, plasma fibrinogen; PLR, platelet to lymphocyte ratio; PSM, propensity score matching; PT, prothrombin time; SMD, standardized mean difference; TBIL, total bilirubin.

^a^ Including those intraoperatively and postoperatively (up to 7 days after surgery). Morphine 30 mg (per os) = morphine 10 mg (iv) = sufentanil 10 μg (iv) = fentanyl 100 μg (iv) = remifentanil 100 μg (iv) = 100 mg tramadol (iv) = tramadol 200 mg (per os) = oxycodone 15 mg (per os) = dezocine 10 mg (iv) = pethidine 100 mg (iv).

### 3.2 Primary analysis

Among the entire study population, perioperative ischemic stroke occurred in 484 patients within 30 days of surgery. Of these events, 424 (0.2%) occurred in the non‐CHD group and 60 (0.7%) occurred in the CHD group, and unadjusted logistic regression analysis showed that CHD was significantly associated with the risk of perioperative ischemic stroke within 30 days of surgery (OR 3.7943; 95% CI, 2.865–4.934; *p* < 0.001; Table 2). Moreover, CHD was significantly associated with an increased risk of perioperative ischemic stroke within 30 days after noncardiac surgery after adjustment for patient‐related confounders (OR 1.5233; 95% CI, 1.124–2.033; *p* = 0.005), surgery‐related confounders (OR 4.0022; 95% CI, 3.006–5.236; *p* = 0.005), as well as for all 26 prospectively defined confounders (OR 1.8415; 95% CI, 1.355–2.463; *p* < 0.001) (Table 2). The complete data are detailed in Table S3.

**TABLE 2:** Logistic regression and propensity score analysis of the association between CHD and perioperative ischemic stroke.

Analysis method	OR	95% CI	*p* value
Logistic regression analysis (*N* = 221,541)			
Model 1 (unadjusted)^a^	3.7943	2.865–4.934	<0.001
Model 2 (patient‐related confounders adjusted)^b^	1.5233	1.124–2.033	0.005
Model 3 (surgery‐related confounders adjusted)^c^	4.0022	3.006–5.236	0.005
Model 4 (fully adjusted)^d^	1.8415	1.355–2.463	<0.001
Propensity score analysis			
PS matching (*N* = 23,484)^e^	1.8150	1.254–2.619	0.001

Abbreviations: CI, confidence interval; OR, odds ratio; PS, propensity score.

^a^ Model 1 was a univariable crude model.

^b^ Model 2 included age, sex, BMI, ASA Class, arrhythmia, COPD, chronic kidney disease, preoperative β blockers, preoperative Hb, preoperative ALB, preoperative TBIL, preoperative PT, preoperative NLR, preoperative PLR, preoperative PF, preoperative FAR.

^c^ Model 3 included emergency surgery, surgery type, malignant tumor, surgery length, estimated blood loss, preoperative MAP, blood products depot, colloids infusion, crystalloids infusion, and morphine equivalents.

^d^ Model 4 included all the above confounders. Full results are displayed in Table S3.

^e^ 15,656 Pairs were matched by propensity score. Full results are displayed in Table S4.

### 3.3 PSM analysis and adjustment

To minimize differences, we performed 2:1 matching of patients without CHD:with CHD for age, BMI, ASA class, preoperative β‐blockers, and surgery type. This resulted in 7828 patients in the CHD group and 15,656 patients in the non‐CHD group, and the K‐densities were similar between the two groups (Figure 2A,B). Logistic regression analysis of the PSM cohorts confirmed a significant association between CHD and the risk of perioperative ischemic stroke (OR 1.8150; 95% CI, 1.254–2.619; *p* = 0.001; Table 2). The complete data are detailed in Table S4.

### 3.4 Subgroup analysis

**FIGURE 3:**
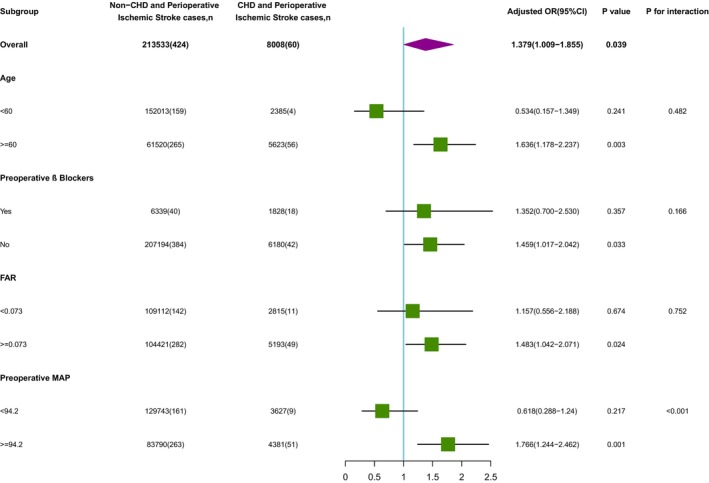
Subgroup analysis of the association between CHD and the risk of perioperative ischemic stroke. CHD, coronary heart disease; FAR, fibrinogen to albumin ratio; MAP, mean arterial pressure; OR, odds ratio.

To explore whether there were differences in the association between CHD and perioperative ischemic stroke in different populations, we next performed subgroup analyses according to patient status for β‐blockers, FAR, age, and preoperative MAP (Figure 3). For age (<60 years vs. ≥60 years; *p*‐interaction = 0.482), preoperative β‐blockers (receive preoperative β‐blockers vs. did not receive preoperative β‐blockers; *p*‐interaction = 0.166), FAR (<0.073 vs. ≥0.073; *p*‐interaction = 0.752). None of the above variables significantly changed the relationship between CHD and perioperative ischemic stroke. We found an interaction effect only in the preoperative MAP subgroup (MAP <94.2 mmHg vs. MAP ≥94.2 mmHg; *p*‐interaction <0.001); it is worth mentioning that the risk of perioperative ischemic stroke was significant among patients with preoperative MAP ≥94.2 mmHg (OR (95% CI): 2.362 (1.676, 3.273); *p* < 0.001), whereas there was no significant association for patients with preoperative MAP <94.2 mmHg (OR (95% CI): 0.822 (0.376, 1.586); *p* = 0.587).

## 4 DISCUSSION

Perioperative stroke is a serious complication of surgery, which is independently associated with increased cognitive dysfunction, physical disability, and mortality.^20^ CHD is one of the most common cardiovascular diseases, and patients with CHD suffer from high morbidity and mortality.^21^ Patients with CHD are generally considered at increased risk for serious peri‐operative complications, including stent thrombosis and myocardial infarction, as well as increased risk for peri‐operative mortality.^22^, ^23^, ^24^, ^25^, ^26^, ^27^, ^28^ While CHD has been identified as an independent risk factor for ischemic stroke,^29^, ^30^, ^31^ whether CHD is remarkably related to the risk of perioperative ischemic stroke has not been reported.

In this study, we evaluated the incidence of perioperative ischemic stroke in 221,541 patients undergoing noncardiac surgery. The overall incidence of perioperative ischemic stroke in our study was 0.22% (484 patients), which is consistent with previous reports of the incidence of these events.^2^, ^3^ However, the risk of perioperative ischemic stroke was remarkably higher among patients with CHD (0.7%) compared to patients without CHD (0.2%). To our knowledge, this is the first large‐sample study of the effect of CHD on the risk of perioperative ischemic stroke, although there have been a few studies evaluating the incidence of perioperative ischemic stroke in patients with CHD. A prospective study of 1134 patients with coronary stents reported that the risk of postoperative stroke in noncardiac surgery patients was 0.09%.^27^ In a retrospective study of 666 patients with coronary stents, the incidence of perioperative stroke was 0.3%.^28^ We note that both of these studies found a lower incidence of perioperative stroke compared to our study, which may result from several differences in how the studies were designed. For example, our study included patients with all kinds of CHD, whereas the two previous studies only included patients who had coronary stents. Also, the first study included short operations, such as endoscopic treatment, in which the risk of perioperative stroke is extremely low, and the second study had a relatively small sample size. In addition, the previous studies only evaluated stroke as a secondary outcome in patients with CHD undergoing surgery, and they did not systematically analyze the correlation between CHD and the risk of perioperative stroke. Our data indicate that CHD is an independent hazard factor for perioperative ischemic stroke and future multicenter, large, prospective studies are needed to verify the incidence of perioperative stroke in this patient population.

Our unadjusted analysis found that CHD is significantly associated with an increased risk of perioperative ischemic stroke. However, this was a very large and diverse patient population with many factors that could contribute to stroke risk. To confirm the relationship between CHD and perioperative stroke, we prospectively defined 26 patient‐, and surgery‐related confounding factors and developed four adjusted models. Strikingly, regression analysis using models adjusted for patient‐related confounders, surgery‐related confounders, and all 26 confounders, each model confirmed a statistically significantly increased risk of perioperative ischemic stroke within 30 days of noncardiac surgery in patients with CHD compared with patients without CHD. Although our study does not evaluate the mechanism by which CHD contributes to risk of perioperative ischemic stroke, one possible explanation is that CHD is characterized by coronary atherosclerosis. Atherosclerosis is a diffuse process that occurs in multiple vascular beds, including carotid, coronary, aortic, and peripheral vessels, and a previous study found that atherosclerosis of the ascending aorta was an independent predictor of perioperative stroke.^32^

We used PSM to identify 2:1 matched non‐CHD:CHD cohorts with similar K‐densities that would allow us to verify the correlation between CHD and increased risk of perioperative ischemic stroke, which again confirmed a remarkable correlation between CHD and increased risk of perioperative ischemic stroke. Further, we evaluated the relationship between CHD and risk of perioperative stroke in subgroups defined by characteristics that have been associated with the risk of CHD complications, including advanced age, β‐blockers, FAR, and preoperative MAP.^33^, ^34^, ^35^, ^36^ A range of interventions targeting hypertension have been reported in the literature to be effective in reducing the risk of CHD complications.^33^, ^34^ In this study, a subgroup analysis based on MAP level showed that patients with CHD and preoperative MAP ≥94.2 mmHg had a much higher risk of perioperative ischemic stroke, suggesting that strict blood pressure control could help reduce the risk of these adverse events. A previous study showed that preoperative concomitant use of statin and β‐blocker drugs significantly decreased the risk of perioperative stroke after coronary‐artery‐bypass‐grafting surgery, whereas taking statin or β‐blocker drugs alone did not affect the risk of perioperative stroke.^36^ And a POISE trial found that the use of β‐blockers was associated with a lower risk of atrial fibrillation but a higher risk of perioperative stroke (due largely to an increase in hypotension) and of overall mortality in patients undergoing noncardiac surgery.^37^ Consistent with the previous study above, our study found that preoperative use of β‐blocker therapy alone did not affect the risk of perioperative ischemic stroke. Moreover, another study showed that, in patients with abnormal FAR, targeted anticoagulant therapy, dietary adjustment, and intravenous protein supplementation could improve patient hypercoagulability and nutritional status, leading to improved prognosis and prolonged survival.^35^ In contrast, our subgroup analysis showed that FAR level did not affect the risk of perioperative ischemic stroke in patients with CHD. Taken together, our data suggest that age, preoperative β‐blockers, and FAR did not affect the association between CHD and perioperative ischemic stroke, whereas preoperative MAP ≥94.2 mmHg in patients with CHD had a much higher risk of perioperative ischemic stroke, and preoperative control of blood pressure in patients with CHD should be considered to reduce the risk of perioperative ischemic stroke in patients with CHD.

An important strength of our study is the large patient sample. Owing to the low rate of perioperative ischemic stroke, we evaluated a very large retrospective database of 376,933 surgical patients to identify sufficient perioperative ischemic stroke events to enable statistical analysis. Also, our study integrated preoperative, intraoperative, and postoperative data into this retrospective database for accurate effect size assessment. Finally, we performed sensitivity analyses, including PSM analysis and subgroup analyses, which validated the robustness of our findings.

Our study has several potential limitations that should be considered when interpreting the findings. First, because this was a retrospective study, we cannot draw conclusions regarding causality; thus, large, randomized clinical trials are needed to find out whether there may be a causal relationship between CHD and the risk of perioperative ischemic stroke. Second, our data were obtained from a single hospital, and our findings may therefore not be generalizable to other hospitals or regions. Third, although we considered confounding factors in our analyses, the presence of residual and unmeasured potential confounders cannot be completely excluded in observational studies. Fourth, despite our efforts, we were not able to obtain all records regarding preoperative drug treatment in patients with CHD, and it is possible that there were other therapies for which we should have adjusted our analyses. Finally, we did not investigate the relationship between CHD and long‐term survival outcomes of perioperative ischemic stroke, and this is a clinically relevant finding that should be evaluated in future studies.

## 5 CONCLUSION

CHD is significantly associated with an increased risk of perioperative ischemic stroke, and CHD was identified as an independent risk factor for perioperative ischemic stroke in our large retrospective cohort study of noncardiac surgery patients. Strict control of blood pressure may reduce the risk of perioperative ischemic stroke in patients with CHD. Future large, randomized clinical trials are needed to figure out a causal relationship between CHD and the risk of perioperative ischemic stroke, as well as to evaluate the impact of CHD on the long‐term survival of perioperative ischemic stroke patients.

## AUTHOR CONTRIBUTIONS

YM and WM conceived and designed the study. RW, FL, PL, YS, LM, YL, and MS contributed to data extraction and acquisition. RW, HW, and SL drafted the manuscript. RW, SL, and YM analyzed and interpreted the data. YM and WM supervised the study. WH helped to revise the manuscript. YM was the guarantor of this study, had full access to all the study data, and took responsibility for the integrity and accuracy of the data analyses. All authors contributed to the article and approved the submitted version.

## FUNDING INFORMATION

This work was supported by grants from the Capital Health Research and Development of Special (No. 2022‐4‐5025), the National Key Research and Development Program of China (No. 2018YFC2001901), and the National Natural Science Foundation of China (Nos. 82171464, 81801193, 82371469).

## CONFLICT OF INTEREST STATEMENT

None.

## Supporting information


**Table S1.**.

## Data Availability

The data underlying this article will be shared on reasonable request to the corresponding author.
