# Supplementary material for: Coronary heart disease increases the risk of perioperative ischemic stroke after noncardiac surgery: A retrospective cohort study
Source: CNS Neurosci Ther. 2024 Aug 26;30(8):e14912. doi: 10.1111/cns.14912 (PMC11345749; doi:10.1111/cns.14912)
Supplement: Supplementary file 1 — Table S1.. [file CNS-30-e14912-s001.docx]

SUPPLEMENTAL MATERIAL

**Supplementary Table S1.** STROBE Statement—Checklist of Items that Should Be Included in Reports of Cohort Studies

|  | Item No | Recommendation | Page No |
| --- | --- | --- | --- |
| **Title and abstract** | 1 | (*a*) Indicate the study’s design with a commonly used term in the title or the abstract | 2,3 |
|  |  | (*b*) Provide in the abstract an informative and balanced summary of what was done and what was found | 2,3 |
| Introduction | | |  |
| Background/rationale | 2 | Explain the scientific background and rationale for the investigation being reported | 4,5 |
| Objectives | 3 | State specific objectives, including any prespecified hypotheses | 5 |
| Methods | | |  |
| Study design | 4 | Present key elements of study design early in the paper | 5,6 |
| Setting | 5 | Describe the setting, locations, and relevant dates, including periods of recruitment, exposure, follow-up, and data collection | 5,6 |
| Participants | 6 | (*a*) Give the eligibility criteria, and the sources and methods of selection of participants. Describe methods of follow-up | 5,6 |
|  |  | (*b*) For matched studies, give matching criteria and number of exposed and unexposed | 5,6 |
| Variables | 7 | Clearly define all outcomes, exposures, predictors, potential confounders, and effect modifiers. Give diagnostic criteria, if applicable | 6 |
| Data sources/ measurement | 8* | For each variable of interest, give sources of data and details of methods of assessment (measurement). Describe comparability of assessment methods if there is more than one group | 5,6 |
| Bias | 9 | Describe any efforts to address potential sources of bias | 7 |
| (Continued) | | | |
| **Supplementary Table S1** (Continued) | | | |
|  | Item No | Recommendation | Page No |
| Study size | 10 | Explain how the study size was arrived at | 5,6 |
| Quantitative variables | 11 | Explain how quantitative variables were handled in the analyses. If applicable, describe which groupings were chosen and why | 6,7 |
| Statistical methods | 12 | (*a*) Describe all statistical methods, including those used to control for confounding | 6,7,8 |
|  |  | (*b*) Describe any methods used to examine subgroups and interactions | 7 |
|  |  | (*c*) Explain how missing data were addressed | Not applicable |
|  |  | (*d*) If applicable, explain how loss to follow-up was addressed | Not applicable |
|  |  | (*e*) Describe any sensitivity analyses | 7 |
| Results | | |  |
| Participants | 13* | (a) Report numbers of individuals at each stage of study—eg numbers potentially eligible, examined for eligibility, confirmed eligible, included in the study, completing follow-up, and analysed | 5,6 |
|  |  | (b) Give reasons for non-participation at each stage | Not applicable |
|  |  | (c) Consider use of a flow diagram | Figure 1 |
| Descriptive data | 14* | (a) Give characteristics of study participants (eg demographic, clinical, social) and information on exposures and potential confounders | Table 1 |
|  |  | (b) Indicate number of participants with missing data for each variable of interest | Not applicable |
|  |  | (c) Summarise follow-up time (eg, average and total amount) | Not applicable |
| Outcome data | 15* | Report numbers of outcome events or summary measures over time | 8 |
| (Continued) | | | |
| **Supplementary Table S1** (Continued) | | | |
|  | Item No | Recommendation | Page No |
| Main results | 16 | (*a*) Give unadjusted estimates and, if applicable, confounder-adjusted estimates and their precision (eg, 95% confidence interval). Make clear which confounders were adjusted for and why they were included | Table 2, Supplementary Table S3, Supplementary Table S4 |
|  |  | (*b*) Report category boundaries when continuous variables were categorized | 7,8 |
|  |  | (*c*) If relevant, consider translating estimates of relative risk into absolute risk for a meaningful time period | Not applicable |
| Other analyses | 17 | Report other analyses done—eg analyses of subgroups and interactions, and sensitivity analyses | 9,10 |
| Discussion | | |  |
| Key results | 18 | Summarise key results with reference to study objectives | 10-14 |
| Limitations | 19 | Discuss limitations of the study, taking into account sources of potential bias or imprecision. Discuss both direction and magnitude of any potential bias | 14,15 |
| Interpretation | 20 | Give a cautious overall interpretation of results considering objectives, limitations, multiplicity of analyses, results from similar studies, and other relevant evidence | 15 |
| Generalisability | 21 | Discuss the generalisability (external validity) of the study results | Not applicable |
| Other information | | |  |
| Funding | 22 | Give the source of funding and the role of the funders for the present study and, if applicable, for the original study on which the present article is based | 17 |

**Supplementary Table S2.** ICD-9/10 Diagnosis Codes for Ischemic Stroke

| Ischemic stroke | ICD-9/ICD-10 | 433.X1/I63.X | Occlusion and stenosis of precerebral arteries with cerebral infarction |
| --- | --- | --- | --- |
|  | ICD-9 | 434.X1 | Occlusion of cerebral arteries with cerebral infarction |
|  | ICD-9/ICD-10 | 437.1/I67.81,  I67.89 | Other generalized ischemic cerebrovascular disease |
|  | ICD-9/ICD-10 | 437.9/I67.9 | Unspecified cerebrovascular disease |

**Supplementary Table S3**. Univariate and Multivariate Logistic Regression Analysis for Perioperative Stroke in Model 4.

| **Variables** | **Logistic regression analysis** | | |
| --- | --- | --- | --- |
|  | **OR** | **CI** | **P** |
| **CHD (Yes vs No)** | 1.8415 | 1.355-2.463 | < 0.001 |
| **Sex (male vs female)** | 1.0302 | 0.835-1.271 | 0.7811 |
| **Age [65,75)** | 3.0724 | 2.482-3.797 | < 0.001 |
| **Age [75,81)** | 3.7046 | 2.626-5.138 | < 0.001 |
| **Age [81,100]** | 3.0910 | 1.590-5.480 | 0.0003 |
| **BMI** | 1.0047 | 0.978-1.032 | 0.7326 |
| **ASA physical status (%)** |  |  |  |
| Class I |  |  |  |
| Class II | 1.8777 | 1.193-3.154 | 0.0106 |
| Class III | 3.3390 | 2.039-5.782 | < 0.001 |
| **Preoperative β blockers (Yes vs No)** | 1.8274 | 1.343-2.445 | < 0.001 |
| **Preoperative Hb** | 1.0001 | 0.994-1.007 | 0.9861 |
| **Preoperative ALB** | 0.9696 | 0.944-0.996 | 0.02303 |
| **Preoperative TBIL** | 1.0001 | 0.997-1.003 | 0.9277 |
| **Preoperative PT** | 1.0576 | 0.984-1.119 | 0.0850 |
| **Preoperative NLR** | 1.0090 | 0.984-1.031 | 0.4481 |
| **Preoperative PLR** | 1.0009 | 1-1.002 | 0.1013 |
| **Preoperative PF** | 1.3172 | 0.997-1.765 | 0.0586 |
| **Preoperative FAR** | 1.1522 | 0.831-1.585 | 0.3890 |
| **Chronic kidney disease (Yes vs No)** | 1.1648 | 0.588-2.075 | 0.6322 |
| **COPD (Yes vs No)** | 0.7189 | 0.221-1.702 | 0.5154 |
| **Arrhythmia (Yes vs No)** | 1.1085 | 0.873-1.393 | 0.3874 |
| **Emergency surgery (Yes vs No)** | 0.3541 | 0.247-0.517 | < 0.001 |
| **Surgery type** |  |  |  |
| Orthopaedic surgery | 0.4028 | 0.277-0.582 | < 0.001 |
| Intraperitoneal surgery | 0.5378 | 0.273-0.971 | 0.0530 |
| Gynecologic surgery | 0.9242 | 0.517-1.564 | 0.7789 |
| Oral surgery | 3.8617 | 2.864-5.224 | < 0.001 |
| Neurosurgical surgery | 0.4625 | 0.255-0.796 | 0.0076 |
| Thoracic surgery | 0.6605 | 0.475-0.916 | 0.0132 |
| **Malignant tumor (Yes vs No)** | 0.8399 | 0.667-1.056 | 0.1359 |
| **Surgery length, min** | 1.0017 | 1.001-1.003 | 0.0011 |
| **Estimated blood loss** |  |  |  |
| (200,400] | 1.0691 | 0.807-1.4 | 0.6338 |
| (400,800] | 0.9220 | 0.635-1.316 | 0.6624 |
| >800 | 1.1449 | 0.712-1.811 | 0.5692 |
| (Continued) | | | |
| **Supplementary Table S3** (Continued) | | | |
| **Variables** | **Logistic regression analysis** | | |
|  | **OR** | **CI** | **P** |
| **Preoperative MAP** | 1.0329 | 1.025-1.041 | < 0.001 |
| **Blood products depot (Yes vs No)** | 0.9901 | 0.722-1.347 | 0.9500 |
| **Crystalloids infusion, ml/kg/min** |  |  |  |
| (7,10] | 0.9601 | 0.76-1.209 | 0.7306 |
| (10,71] | 0.9827 | 0.746-1.29 | 0.9007 |
| **Colloids infusion, ml/kg/min** |  |  |  |
| (1,4] | 1.3212 | 1.039-1.691 | 0.0248 |
| (4,40] | 1.3260 | 0.985-1.784 | 0.0624 |
| **Morphine equivalents** | 1.0009 | 0.999-1.003 | 0.3792 |

| Abbreviations: CHD, coronary heart disease; BMI, body mass index; ASA, American Society of Anesthesiologists; Hb, hemoglobin; ALB, albumin; TBIL, total bilirubin; NLR, neutrophil-lymphocyte ratio; PLR, platelet to lymphocyte ratio; PT, prothrombin time; PF, plasma fibrinogen; FAR, fibrinogen to albumin ratio; COPD, chronic obstructive pulmonary disease; MAP, mean arterial pressure |
| --- |

**Supplementary Table S4**. Univariate Logistic Regression Analysis for Perioperative Stroke in the PS Matched Cohort.

| **Variables** | **Logistic regression analysis** | | |
| --- | --- | --- | --- |
|  | **OR** | **CI** | **P** |
| **CHD (Yes vs No)** | 1.8150 | 1.254-2.619 | 0.0015 |
| **Sex (male vs female)** | 1.1540 | 0.771-1.725 | 0.4850 |
| **Age [65,75)** | 2.5088 | 1.603-4.003 | < 0.001 |
| **Age [75,81)** | 2.8304 | 1.553-5.088 | 0.0006 |
| **Age [81,100]** | 3.0884 | 1.244-6.919 | 0.0092 |
| **BMI** | 1.0058 | 0.952-1.062 | 0.8362 |
| **ASA physical status (%)** |  |  |  |
| Class I |  |  |  |
| Class II | 511459.5 | 0.076-3.82135373027098e+86 | 0.9783 |
| Class III | 776783.3 | 2.04092114540934e+45-4.30105679103281e+90 | 0.9776 |
| **Preoperative β blockers (Yes vs No)** | 1.4106 | 0.924-2.11 | 0.1014 |
| **Preoperative Hb** | 0.9915 | 0.98-1.003 | 0.1607 |
| **Preoperative ALB** | 0.9635 | 0.914-1.016 | 0.1678 |
| **Preoperative TBIL** | 0.9949 | 0.983-1.002 | 0.2641 |
| **Preoperative PT** | 1.0080 | 0.861-1.122 | 0.9018 |
| **Preoperative NLR** | 1.0350 | 0.998-1.066 | 0.0370 |
| **Preoperative PLR** | 1.0003 | 0.998-1.002 | 0.7799 |
| **Preoperative PF** | 1.2004 | 0.706-2.162 | 0.5204 |
| **Preoperative FAR** | 1.0670 | 0.539-2.073 | 0.8495 |
| **Chronic kidney disease (Yes vs No)** | 2.1891 | 0.929-4.527 | 0.0498 |
| **COPD (Yes vs No)** | 0.7726 | 0.124-2.555 | 0.7251 |
| **Arrhythmia (Yes vs No)** | 1.4760 | 0.974-2.195 | 0.0598 |
| **Emergency surgery (Yes vs No)** | 0.7091 | 0.332-1.654 | 0.3988 |
| **Surgery type** |  |  |  |
| Orthopaedic surgery | 0.5755 | 0.285-1.164 | 0.1229 |
| Intraperitoneal surgery | 0.0000 | 0-0 | 0.9740 |
| Gynecologic surgery | 1.1499 | 0.254-3.668 | 0.8323 |
| Oral surgery | 5.3579 | 2.8-10.32 | < 0.001 |
| Neurosurgical surgery | 0.7829 | 0.29-1.952 | 0.6117 |
| Thoracic surgery | 0.6917 | 0.36-1.328 | 0.2665 |
| (Continued) | | | |
| **Supplementary Table S4** (Continued) | | | |
| **Variables** | **Logistic regression analysis** | | |
|  | **OR** | **CI** | **P** |
| **Malignant tumor (Yes vs No)** | 0.8121 | 0.507-1.301 | 0.3861 |
| **Surgery length, min** | 1.0016 | 0.999-1.004 | 0.1889 |
| **Estimated blood loss** |  |  |  |
| (200,400] | 0.6666 | 0.334-1.223 | 0.2164 |
| (400,800] | 0.8470 | 0.373-1.758 | 0.6726 |
| >800 | 1.4869 | 0.597-3.509 | 0.3779 |
| **Preoperative MAP** | 1.0323 | 1.016-1.049 | 0.0001 |
| **Blood products depot (Yes vs No)** | 0.9464 | 0.503-1.724 | 0.8605 |
| **Crystalloids infusion, ml/kg/min** |  |  |  |
| (7,10] | 0.8922 | 0.557-1.409 | 0.6285 |
| (10,71] | 0.8765 | 0.511-1.487 | 0.6278 |
| **Colloids infusion, ml/kg/min** |  |  |  |
| (1,4] | 1.6915 | 1.019-2.919 | 0.0491 |
| (4,40] | 2.3834 | 1.314-4.397 | 0.0046 |
| **Morphine equivalents** | 1.0030 | 0.999-1.007 | 0.1608 |

| Abbreviations: PS, propensity score; CHD, coronary heart disease; BMI, body mass index; ASA, American Society of Anesthesiologists; Hb, hemoglobin; ALB, albumin; TBIL, total bilirubin; NLR, neutrophil-lymphocyte ratio; PLR, platelet to lymphocyte ratio; PT, prothrombin time; PF, plasma fibrinogen; FAR, fibrinogen to albumin ratio; COPD, chronic obstructive pulmonary disease; MAP, mean arterial pressure |
| --- |

**Supplementary Table S5. Abbreviation**

| **Abbreviation** | **Full English name** |
| --- | --- |
| CHD | Coronary heart disease |
| OR | Odds ratio |
| CI | Confidence interval |
| PSM | Propensity score matching |
| FAR | Fibrinogen-to-albumin ratio |
| MAP | Mean arterial pressur |
| TIA | Transient ischemic attack |
| ASA | American Society of Anesthesiologists |
| DM | Diabetes mellitus |
| BMI | Body mass index |
| Hb | Hemoglobin |
| ALB | Albumin |
| TBIL | Total bilirubin |
| PT | Prothrombin time |
| NLR | Neutrophil-to-lymphocyte ratio |
| PF | Plasma fibrinogen |
| PLR | Platelet-to-lymphocyte ratio |
| COPD | Chronic obstructive pulmonary disease |
| SMD | Standardized mean difference |
